# Management and consequences of postoperative fluctuations in plasma sodium concentration after pediatric brain tumor surgery in the sellar region: a national cohort analysis

**DOI:** 10.1007/s11102-018-0886-2

**Published:** 2018-04-05

**Authors:** R. W. J. Kruis, A. Y. N. Schouten-van Meeteren, M. J. J. Finken, W. Oostdijk, A. S. P. van Trotsenburg, A. M. Boot, H. L. Claahsen-van der Grinten, E. J. van Lindert, K. S. Han, E. W. Hoving, E. M. C. Michiels, H. M. van Santen

**Affiliations:** 1 Department of Pediatric Endocrinology, University Medical Center Utrecht – Wilhelmina Children’s Hospital, Utrecht, The Netherlands; 2 Department of Pediatric Oncology, Academic Medical Center – Emma Children’s Hospital, Amsterdam, The Netherlands; 3 Department of Pediatric Endocrinology, VU University Medical Center, Amsterdam, The Netherlands; 4 Department of Pediatric Endocrinology, Leiden University Medical Center – Willem-Alexander Children’s Hospital, Leiden, The Netherlands; 5 Department of Pediatric Endocrinology, Academic Medical Center – Emma Children’s Hospital, Amsterdam, The Netherlands; 6 Department of Pediatric Endocrinology, University Medical Center Groningen – Beatrix Children’s Hospital, University of Groningen, Groningen, The Netherlands; 7 Department of Pediatric Endocrinology, Radboud University Medical Center – Amalia Children’s Hospital, Nijmegen, The Netherlands; 8 Department of Pediatric Neurosurgery, Radboud University Medical Center – Amalia Children’s Hospital, Nijmegen, The Netherlands; 9 Department of Pediatric Neurosurgery, University Medical Center Utrecht – Wilhelmina Children’s Hospital, Utrecht, The Netherlands; 10 Department of Pediatric Neurosurgery, University Medical Center Utrecht – Princess Máxima Center, Utrecht, The Netherlands; 11 Department of Pediatric Oncology, Erasmus University Medical Center – Sophia Children’s Hospital, Rotterdam, The Netherlands; 12 Wilhelmina Children’s Hospital, University of Utrecht, Postal adress KC.03.063.0, PO Box 85090, 3508 AB Utrecht, The Netherlands

**Keywords:** Child, Plasma sodium concentration, Diabetes insipidus, Brain neoplasm, Neurological effect, Neurological consequence

## Abstract

**Purpose:**

Severe fluctuations in plasma sodium concentration and plasma osmolarity, including central diabetes insipidus (CDI), may have significant influence on postoperative morbidity and mortality after pediatric brain tumor surgery.The aim of this study was to describe the frequency, severity and neurological consequences of these fluctuations in pediatric brain tumor survivors.

**Methods:**

A retrospective, multi-institutional chart review was conducted among all children who underwent brain tumor surgery in the sellar or suprasellar region in seven university hospitals in the Netherlands between January 2004 and December 2013.

**Results:**

Postoperative CDI was observed in 67.5% of 120 included children. Fluctuations of plasma sodium concentration ≥ 10 mmol/L/24 h during the first ten postoperative days were seen in 75.3% of patients with CDI, with a maximum delta of 46 mmol/L/24 h. When compared to patients without CDI, altered mental status occurred more frequently in patients with postoperative CDI (5.1 vs. 23.5% respectively, p = 0.009). Low plasma sodium concentration was related to altered mental status and the occurrence of seizures. Frequency and severity of fluctuations in plasma sodium concentration during the first ten postoperative days were significantly higher in patients with permanent CDI at last follow-up than in patients with transient CDI or without CDI (p = 0.007).

**Conclusion:**

Postoperative CDI is a common complication after pediatric brain tumor surgery in the sellar or suprasellar region. Extreme plasma sodium concentrations and large intra-day fluctuations still occur and seem to influence the postoperative neurological course. These results illustrate the need for intensive monitoring in a highly experienced center.

## Introduction

Tumors in the sellar or suprasellar region account for approximately 10% of all pediatric brain tumors [1]. Craniopharyngiomas account for the majority of sellar tumors in children, representing 1.2–4% of all pediatric intracranial tumors [2, 3]. Although central diabetes insipidus (CDI) can be the presenting symptom of sellar tumors, most often it occurs as a complication of the surgical treatment, with incidences ranging from 10 to 60% [4–6]. Patients with postoperative CDI have a transient or permanent lack of antidiuretic hormone (ADH), caused by perioperative manipulation or damage to the neurohypophyseal system. CDI in the postoperative period may present with a triphasic response in the sodium and fluid balance [CDI, followed by the syndrome of inappropriate ADH secretion (SIADH), followed again by CDI]. This triphasic response may be explained by an axonic shock and instability of the posterior pituitary to secrete ADH, followed by leakage of ADH from the distorted neurons. Exhaustion of all ADH results in permanent CDI [7]. During the first hours postoperatively, it may be difficult to distinguish between CDI and polyuria caused by perioperative administration of large volumes of fluids. In the second phase of the triphasic response with oliguria and hyponatremia, it can be challenging to differentiate inappropriate secretion of ADH (endogenous or exogenous) from cerebral salt wasting or the administration of hypotonic fluids. The occurrence of postoperative CDI, with or without the triphasic response, may lead to significant fluctuations in plasma sodium concentration. Abnormal or steep changes in plasma sodium concentration may have significant influence on postoperative morbidity and mortality [8–10]. Steep decline in plasma sodium concentration (hyponatremia) is known to cause seizures, encephalopathy, and cerebral edema, which are potential sources of secondary brain injury among neurosurgical patients [8]. Severe hypernatremia occurs when fluid replacement is insufficient to compensate for the water loss and/or exogenous sodium substitution. Neurosurgical pediatric patients may have altered or absent awareness of thirst in the direct postoperative period, which makes them vulnerable to developing severe hypernatremia [9]. Acquired hypernatremia appears to signal higher risk of mortality in children with severe traumatic brain injury and has been associated with a higher level of care needed by survivors of severe traumatic brain injury at the time of discharge [10]. For these reasons, it is important to monitor the sodium and fluid balance closely in patients who underwent tumor surgery in the sellar region. The primary aim of our study was to evaluate the frequency, severity and neurological consequences of postoperative fluctuations in plasma sodium concentration in children with (supra)sellar lesions. Secondary, we aimed to disclose the risk factors for developing severe fluctuations in plasma sodium concentration in the early postoperative period (within 10 days after surgery).

## Patients and methods

### Population

A national retrospective cohort was selected from all children diagnosed with a sellar or suprasellar tumor who underwent neurosurgery in the Netherlands between January 2004 and December 2013. Patients were recruited from the seven university hospitals in the Netherlands that offered treatment to children diagnosed with such a tumor. Patients were identified using patient registrations from the departments of Pediatric Neurosurgery, Pediatric Oncology or Pediatric Endocrinology. The lists with selected patients were handsearched by RK together with the local physician to check whether the reported diagnosis and inclusion was correct. Inclusion criteria for this study were (1) diagnosis of a sellar or suprasellar tumor; (2) initial surgery (transcranial or transsphenoidal approach) for tumor resection (gross total or partial resection) or biopsy; (3) age of 18 years or younger at the time of surgical intervention; (4) initial surgery performed in the period from January 2004 to December 2013. Patients were excluded if data on plasma sodium concentration during the first ten postoperative days were not available or if the information from their medical charts was insufficient to determine whether they had experienced postoperative CDI or not. The study was approved by the Medical Ethical Committee of the Wilhelmina Children’s Hospital, Utrecht UMCU, The Netherlands. All collected data were anonymized. Based on these criteria, 130 children were eligible for inclusion. Ten children were excluded from further analysis, because postoperative data on plasma sodium concentration or fluid balance were lacking. In total, 120 patients (70 female and 50 male) were analyzed.

### Data collection

Data at initial tumor surgery were gathered on gender, age, presenting symptoms and findings [seizures, visual impairment, obesity, growth retardation, motor deficit, pre-operative CDI or (pan)hypopituitarism], duration of the presenting symptom at the time of diagnosis, tumor type, surgical data (type of surgery, surgical approach), plasma sodium concentration during the first ten postoperative days (highest and lowest plasma sodium concentration per day, highest and lowest plasma sodium concentration during the first ten postoperative days, delta plasma sodium concentration per 24 h, largest delta plasma sodium concentration per 24 h, total amount of fluctuations in plasma sodium concentration ≥ 10 mmol/L/24 h), postoperative course [occurrence of CDI, transient or permanent CDI, occurrence of hyponatremia, occurrence of the triphasic response, administration of desmopressin pre- and post-operative, length of stay at the intensive care unit (ICU)], short-term neurological consequences possibly related to fluctuations in plasma sodium concentration (epileptic seizures, altered mental status), short-term complications probably not related to fluctuations in plasma sodium concentration, total number of resections per patient, adjuvant treatment (radiotherapy, chemotherapy), age at last follow-up, duration of follow-up, long-term neurological consequences (headache, seizures, visual impairment, motor deficit, cognitive dysfunction, sleeping problems, tiredness), long-term endocrine disorders [prevalence of permanent CDI, (pan)hypopituitarism, obesity] and mortality were collected.

### Definitions

CDI was defined as documented polyuria in combination with high plasma sodium concentration (> 145 mmol/L) or documentation of the diagnosis CDI in the medical chart. CDI was classified as transient when patients did not require treatment with desmopressin anymore at time of hospital discharge. Early postoperative CDI was defined as the occurrence of CDI within ten postoperative days, late postoperative CDI was defined as the presence of permanent CDI at last moment of follow-up. Syndrome of inappropriate ADH secretion (SIADH) was defined as documented oliguria in combination with low plasma sodium concentration (< 135 mmol/L). The triphasic response was specified as the period of CDI in the early postoperative period preceded by a hyponatremic phase again preceded by a phase of polyuria. Short-term neurological consequences possibly related to fluctuations in plasma sodium concentration were defined as the presence of epileptic seizures or altered mental status (Glasgow Coma Scale score < 15 or sleepiness/reduced alertness described in the clinical records) during the first ten postoperative days. Relevant postoperative complications probably occurring independent of fluctuations in plasma sodium concentration were defined as the presence of infections (meningitis, drain infection, central venous catheter infection, septicemia, pneumonia, erysipelas, cellulitis, fever of unknown origin treated with antibiotics), anemia requiring (multiple) blood transfusions or hyperglycaemia requiring insulin therapy. Long-term neurological consequences were defined as headache, epileptic seizures (recent seizure or patient using anti-epileptic medication), visual impairment, motor deficit (decreased motor skills or muscle strength objectified by neurological examination), cognitive dysfunction, sleeping problems or fatigue (as reported in the medical chart) at the last moment of follow-up. Cognitive deficits as poor concentration, memory problems and reduced processing speed were extracted from the clinical records as documented by the treating physician. Having ‘any’ long-term endocrine disorder was defined as the presence of CDI, growth hormone (GH)-deficiency (GHD), hypothyroidism, hypogonadism, adrenocorticotropic hormone (ACTH)-deficiency or obesity at last follow-up. GHD, hypothyroidism, hypogonadism and ACTH-deficiency were scored based on the written information of the treating physician in the medical chart. Panhypopituitarism was defined as a generalized deficit of all of the anterior pituitary hormones. Body mass index (BMI) was calculated based on height and weight documented in the growth chart at last follow-up. Patients with a BMI at or above the 95th percentile were regarded as obese [11].

### Statistical analysis

The SPSS (version 22.0) statistical package was used for analysis. Multivariable logistic regression analysis was performed to identify risk factors by testing associations of clinical characteristics with severe (≥ 10 mmol/L/24 h) fluctuations in the plasma sodium concentration level, adjusted for confounders. Likewise, logistic regression analysis was used to test associations of electrolyte disturbances and other clinical characteristics with short-term and long-term neurological consequences. All p values were based on two-sided testing and p values < 0.05 were considered as statistically significant.

## Results

### Baseline characteristics

The majority of the included patients had been diagnosed with craniopharyngioma (Table 1). Median age at initial surgery was 8.5 years (range 0–18). Type of surgery was mainly partial resection (56.7%) and the transcranial approach was used more often than the transsphenoidal approach (82.5 vs. 17.5%). Children under 7 years of age were all operated using the transcranial approach, while the transsphenoidal approach was used more frequent in adolescents. Thirty-five patients (29.2%) underwent one or more resections after primary tumor surgery because of tumor recurrence or tumor progression. Adjuvant treatment with radiotherapy or chemotherapy was given in 48.3 and 24.2% of patients, respectively. Fifteen patients (12.5%) underwent both radiotherapy and chemotherapy. In nine patients (7.5%), CDI was already present at the time of tumor diagnosis (pre-operative DI). Six patients (5.0%) had been treated with desmopressin before initial tumor surgery, while 82 (68.3%) patients received treatment with desmopressin in the direct postoperative period. Dosage of desmopressin could not be retrieved retrospectively. Median length of stay at the ICU was 2.0 days (range 1–18). Important postoperative complications probably occurring independent of fluctuations in plasma sodium concentration were meningitis (n = 4), septicemia (n = 2), pneumonia (n = 1), cellulitis (n = 1), urinary tract infection (n = 3), fever of unknown origin treated with antibiotics (n = 4), postoperative anemia requiring (multiple) blood transfusion(s) (n = 2) and hyperglycaemia requiring insulin therapy (n = 2). Median duration of follow-up was 5.0 years (range 0.1–10). Mortality during follow-up was 5.0% (n = 6): five patients died from tumor progression and one patient had a rapidly progressive neurological condition that was not related to the original tumor.

**Table 1:** Baseline clinical characteristics of the study population

Patients	n (%)
Total	120 (100.0)
Gender	
Female	70 (58.3)
Pathology report: type of tumor	
Craniopharyngioma or xanthogranuloma	70 (58.3)
Chiasmatic hypothalamic glioma (CHG)	29 (24.2)
Germ cell tumor (GCT)	9 (7.5)
Pituitary tumor^a^	9 (7.5)
Other tumor^b^	3 (2.5)
Median age at initial surgery (years)	8.5 (0–18)
Type of initial surgery	
Gross total resection (GTR)	36 (30.0)
Partial resection	68 (56.7)
Biopsy^c^	13 (10.8)
Surgical approach	
Transcranial	99 (82.5)
Transsphenoidal	21 (17.5)
Total number of resections per patient	
n = 1	85 (70.8)
n = 2	32 (26.7)
n = 3	3 (2.5)
Radiotherapy y/n	58 (48.3)/62 (51.7)
Craniopharyngioma or xanthogranuloma	34 (28.3)
Chiasmatic hypothalamic glioma (CHG)	12 (10.0)
Germ cell tumor (GCT)	7 (5.8)
Pituitary tumor^a^	4 (3.3)
Other tumor^b^	1 (0.8)
Chemotherapy y/n^d^	29 (24.2)/90 (75.0)
Pre-operative diabetes insipidus y/n^e^	9 (7.5)/109 (90.8)
Early postoperative diabetes insipidus y/n	81 (67.5)/39 (32.5)
Permanent diabetes insipidus at last moment of follow-up y/n^e^	80 (66.7)/38 (31.7)
ICU length of stay (days)^f^	2.0 (1–18)
Median duration of follow-up (years)	5.0 (0–10)
Mortality during follow-up	6 (5)

Values are presented as number (%) or median (range)

*Y* yes, *N* no

^a^Adenoma or prolactinoma or Rathke’s cleft cyst

^b^Central neurocytoma or rhabdomyosarcoma or hamartoma

^c^Missing data: 3 (2.5)

^d^Missing data: 1 (0.8)

^e^Missing data: 2 (1.7)

^f^Missing data: 36 (30.0)

### Frequency of postoperative fluctuations in plasma sodium concentration

Early postoperative CDI occurred in 81 patients (67.5%). Seven patients (5.8%) with early postoperative CDI did not have permanent CDI at last moment of follow-up. The postoperative triphasic response was observed in 27 of 81 patients (22.5%). Among these patients, the median onset of the first phase (polyuric) was at day 1 postoperative (range 1–2), median onset of the second phase with oliguria was at day 4 (range 2–8) and median onset of the true CDI/third phase with polyuria was at day 9 (range 4–12). All patients with a postoperative triphasic response had permanent CDI at last follow-up. The triphasic response was also recognized in two out of the nine patients known with initial pre-operative CDI. It was noted that some patients who were already treated with desmopressin before tumor surgery tended to have a temporary reduced need for desmopressin in the postoperative period.

### Severity of fluctuations in plasma sodium concentration

**Fig. 1:**
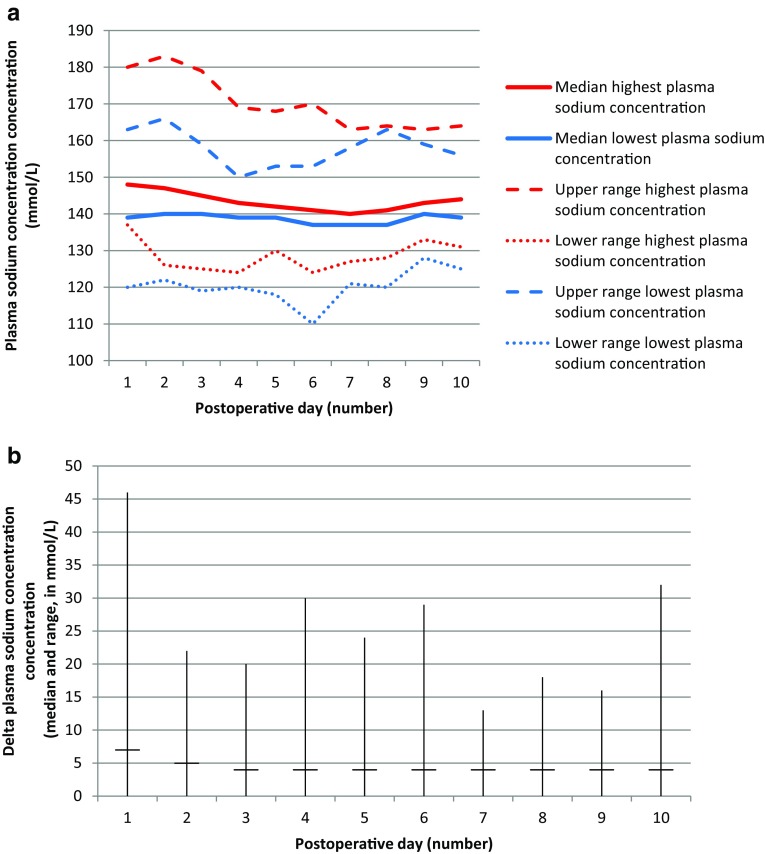
**a** Median highest and median lowest plasma sodium concentration per day (including upper and lower range) during the first 10 days after tumor surgery in the 81 patients with early postoperative DI. **b** Delta plasma sodium concentration per day (including median, upper and lower range) during the first 10 days after tumor surgery in the 81 patients with early postoperative DI

Severe fluctuations of plasma sodium concentration were observed in the 81 patients with early postoperative CDI, ranging from 110 to 183 mmol/L (Fig. 1a, b). The highest plasma sodium concentrations occurred on the first postoperative day. In 1.9% of all measurements in patients with early postoperative CDI, plasma sodium concentrations over 160 mmol/L were found. The lowest plasma sodium concentrations occurred on day 6 until day 8 postoperative. In 1.8% of all measurements in patients with early postoperative CDI, plasma sodium concentrations under 125 mmol/L were found. The maximum delta plasma sodium concentration (defined as the difference between highest and lowest plasma sodium concentration) per day during the first ten postoperative days was 46 mmol/L/day in a patient on the first postoperative day. In 75.3% of patients, fluctuations of plasma sodium concentration ≥ 10 mmol/L/day during the first ten postoperative days were seen, with a median number of two fluctuations ≥ 10 mmol/L/24 h per patient.

### Neurological consequences of fluctuations in plasma sodium concentration

Short-term neurological consequences were observed significantly more frequent in patients with early postoperative CDI compared to patients without early postoperative CDI (32.1 vs. 10.3%, p = 0.007, OR 0.01, 95% CI 0.01–0.02) (Table 2). Altered mental status occurred more often in patients with early postoperative CDI compared to patients without early postoperative CDI (23.5 vs. 5.1%, p = 0.009, OR 0.01, 95% CI 0.01–0.01). At last follow-up, fatigue was observed more frequent in children with early postoperative CDI compared to children without early postoperative CDI (44.4 vs. 23.1%, p = 0.016, OR 0.02, 95% CI 0.02–0.03). Overall lowest plasma sodium concentration was related to the occurrence of short-term neurological consequences [both epileptic seizures and altered mental status, p values of respectively 0.003 (OR 0.77, 95% CI 0.64–0.92) and 0.012 (OR 0.88, 95% CI 0.80–0.97)] (Table 3). All patients (100.0%) with epileptic seizures and 94.7% of patients with an altered mental status experienced fluctuations in plasma sodium concentration ≥ 10 mmol/L/24 h during the first ten postoperative days. No relation was found between changes in the postoperative plasma sodi um concentration and long-term neurological consequences (Table 4).

**Table 2:** Short-term (≤ 10 postoperative days) and long-term neurological consequences in all patients and in patients with or without early postoperative DI

	All patients n (%)	Missing n (%)	Occurrence of early postoperative DI (≤ 10 days)		p value	OR (95% CI)
			Yes n (%)	No n (%)		
Patients	120	–	81	39	–	–
Short-term neurological consequences	30 (25.0)	3 (2.5)	26 (32.1)	4 (10.3)	0.007	0.01 (0.01–0.02)
Epileptic seizure(s)	15 (12.5)	2 (1.7)	13 (16.0)	2 (5.1)	0.084	0.15 (0.14–0.15)
Altered mental status	21 (17.5)	5 (4.2)	19 (23.5)	2 (5.1)	0.009	0.01 (0.01–0.01)
Long-term neurological consequences	104 (86.7)	3 (2.5)	70 (86.4)	34 (87.2)	0.890	1.00 (1.00–1.00)
Headache	31 (25.8)	7 (5.8)	20 (24.7)	11 (28.2)	0.800	0.82 (0.82–0.83)
Epileptic seizure(s)	13 (10.8)	7 (5.8)	11 (13.6)	2 (5.1)	0.141	0.21 (0.20–0.22)
Visual impairment	77 (64.2)	6 (5.0)	51 (63.0)	26 (66.7)	0.889	1.00 (1.00–1.00)
Motor deficit	30 (25.0)	5 (4.2)	22 (27.2)	8 (20.5)	0.392	0.50 (0.49–0.51)
Cognitive dysfunction	39 (32.5)	11 (9.2)	30 (37.0)	9 (23.1)	0.075	0.09 (0.09–0.10)
Sleeping problems	18 (15.0)	9 (7.5)	11 (13.6)	7 (17.9)	0.589	0.79 (0.78–0.80)
Fatigue	45 (37.5)	8 (6.7)	36 (44.4)	9 (23.1)	0.016	0.02 (0.02–0.03)

Values are presented as number (%)

**Table 3:** Multivariate analysis for risk factors to develop short-term neurological consequences in patients with early postoperative DI (n = 81)

	Epileptic seizure(s)			Altered mental status		
	Yes n (%)	p value	OR (95% CI)	Yes n (%)	p value	OR (95% CI)
Patients (n = 81)	13	–	–	19	–	–
Delta plasma sodium concentration ≥ 10 mmol/L/24 h	13 (100.0)	0.998	1.36 × 10^8^ (0.00–)	18 (94.7)	0.410	2.67 (0.26–2.76 × 10^1^)
Overall highest plasma sodium concentration (mmol/L)	151 (140–179)	0.399	0.95 (0.85–1.07)	152 (144–183)	0.050	1.08 (1.00–1.17)
Overall lowest plasma sodium concentration (mmol/L)	121 (110–134)	0.003	0.77 (0.64–0.92)	126 (110–144)	0.012	0.88 (0.80–0.97)
Age at initial surgery (years)	5.0 (0–14)	0.129	0.84 (0.67–1.05)	10.0 (1–18)	0.162	1.12 (0.96–1.30)
Preoperative epileptic seizure(s)	2 (15.4)	0.030	0.04 (0.00–0.72)	2 (10.5)	0.170	0.13 (0.01–2.38)
Surgical approach: transcranial	12 (92.3)	0.394	4.12 (0.16–1.07 × 10^2^)	18 (94.7)	0.182	0.17 (0.01–2.31)
Postoperative complications probably occurring independent of fluctuations in plasma sodium concentration	8 (61.5)	0.167	0.24 (0.03–1.82)	10 (52.6)	0.568	0.69 (0.19–2.47)

Values are presented as number (%) or median (range)

**Table 4:** Multivariate analysis for risk factors to develop long-term neurological consequences in patients with early postoperative DI (n = 81)

	Cognitive dysfunction			Fatigue		
	Yes n (%)	p value	OR (95% CI)	Yes n (%)	p value	OR (95% CI)
Patients (n = 81)	30	–	–	36	–	–
Delta plasma sodium concentration ≥ 10 mmol/L/24 h	27 (90.0)	0.074	5.07 (0.85–30.05)	29 (80.6)	0.439	1.68 (0.45–6.22)
Overall highest plasma sodium concentration (mmol/L)	152 (140–183)	0.201	1.05 (0.97–1.13)	152 (143–183)	0.842	0.99 (0.93–1.06)
Overall lowest plasma sodium concentration (mmol/L)	131.5 (110–145)	0.445	0.97 (0.89–1.06)	132 (110–145)	0.942	1.00 (0.93–1.08)
Total number of resections per patient	1 (1–3)	0.494	1.45 (0.50–4.22)	1 (1–3)	0.758	0.86 (0.33–2.25)
Radiotherapy	22 (73.3)	0.064	0.32 (0.10–1.07)	23 (63.9)	0.106	0.42 (0.15–1.20)
Postoperative complications probably occurring independent of fluctuations in plasma sodium concentration	13 (43.3)	0.711	0.80 (0.24–2.65)	16 (44.4)	0.362	0.62 (0.23–1.72)
Panhypopituitarism at last follow-up	17 (56.7)	0.029	4.68 (1.17–18.79)	27 (75.0)	0.426	0.63 (0.20–1.98)

Values are presented as number (%) or median (range). Long term is defined as measured at last follow-up

### Risk factors for postoperative CDI

Multivariate logistic regression analysis revealed no significant risk factors, in addition to the surgical intervention for the suprasellar tumor, for the occurrence of early postoperative CDI. Permanent CDI at last moment of follow-up was present in 68% of patients. Seven patients (5.8%) without early postoperative CDI finally did present with permanent CDI. Risk factors for developing permanent CDI were early postoperative DI (p = 0.001, OR 0.01, 95% CI 0.00–0.11) and postoperative complications probably occurring independent of fluctuations in plasma sodium concentration complications (p = 0.026, OR 0.06, 95% CI 0.01–0.71). Patients with permanent CDI at last follow-up experienced significantly more fluctuations in plasma sodium concentration ≥ 10 mmol/L/24 h during the first 10 postoperative days than patients with transient CDI and patients without CDI (p = 0.007, OR 26.89, 95% CI 2.43–297.31) (Table 5). No statistically significant differences were found between the various academic medical centers regarding the number of changes in serum sodium concentration > 10 mmol/L within 24 h during the first 10 days postoperative or neurological outcome of the patients.

**Table 5:** Multivariate analysis: pre- and post-operative patient characteristics related to fluctuations in plasma sodium concentration ≥ 10 mmol/L/24 h. during the first ten postoperative days (n = 120)

	p value	OR (95% CI)
Gender	0.822	1.16 (0.31–4.37)
DI at diagnosis	0.564	1.78 (0.25–12.47)
Age at initial surgery (years)	0.300	0.92 (0.79–1.07)
ICU length of stay (days)	0.316	1.14 (0.88–1.48)
Postoperative complications probably occurring independent of fluctuations in plasma sodium concentration	0.698	1.28 (0.36–4.52)
Transient DI during the first ten postoperative days	0.324	5.17 (0.20–135.43)
Permanent DI	0.007	26.89 (2.43–297.31)
Panhypopituitarism at last follow-up	0.837	1.17 (0.27–5.13)
Obesity at last follow-up	0.213	2.36 (0.61–9.08)

*ICU* intensive care unit, *OR* odds ratio, *95% CI* 95% confidence interval

## Discussion

In our large retrospective national cohort study of children operated on sellar or suprasellar tumors and treated in academic centers, a high frequency of CDI was found. The number of clinically relevant fluctuations in plasma sodium concentration was considerable, with plasma sodium levels ranging from 110 to 183 mmol/L and a maximum delta of 46 mmol/L/24 h. These findings illustrate the difficulty in management of postoperative CDI in children, which may partly be due to the low incidence of tumors in the sellar or suprasellar region in children in combination with the occurrence of the unpredictable triphasic response. The results of this study emphasize the need for highly experienced centers taking care of these patients, which was also previously concluded by Edate and Albanese [12]. They suggested that these patients should be referred to specialist centers and managed by designated multidisciplinary teams involving a neuroradiologist, pediatric neurosurgeon, pediatric oncologist and pediatric endocrinologist with access to appropriate specialist pathology and pediatric intensive care facilities. The incidence of postoperative CDI (68%) in our study was high compared to the incidence of 10–63% as described in literature [4–6, 13], possibly explained by the inclusion criteria of our cohort. Radical excision of craniopharygioma, recurrent tumor surgery and transcranial approach have been identified as predictors for early postoperative CDI in children [13–21]. In our study, none of these factors predicted early postoperative CDI. The triphasic response was observed in 23% (n = 27) of patients, with a median day of onset of the first phase on day 1. Remarkably, some patients who had already been diagnosed with pre-operative CDI also experienced a triphasic response after surgery, without need for desmopressin in the phase of SIADH. In these patients, the hyponatriemic phase was most probably caused by overtreatment with desmopressin, excessive fluids and/or perhaps intercurrent cerebral salt wasting [22]. Our data confirm previous findings that all patients with the triphasic response in the postoperative period develop permanent CDI [7]. The patients with permanent CDI showed the most severe fluctuations in plasma sodium concentration during the early postoperative period. This finding suggests that patients with difficulties in management of diabetes insipidus in the early postoperative period are more likely to develop permanent CDI. It was remarkable that all patients with early postoperative CDI were at risk of short-term (altered mental status) and long-term (fatigue) neurological consequences. Especially the steep decline in plasma sodium concentration during the early postoperative course, resulting in hyponatremia, was associated with immediate neurological consequences (epileptic seizures and altered mental status). Our results expand on previous findings by Williams et al. [8], who studied the incidence of postoperative hyponatremia and associated neurological consequences in children with a brain tumor. In their retrospective cohort study of 319 children, hyponatremia occurred in 39 children (12%) and was frequently symptomatic: 21% had seizures and 41% had altered mental status. Also, in this study hyponatremia was associated with more complicated hospital courses (mechanical ventilation, supplemental nutrition), infection and moderate or worse disability by Pediatric Cerebral Performance Category score at discharge. Bohl et al. [23] identified delayed hyponatremia (SIADH) and DI as the most common causes of 30-day unplanned readmission after transsphenoidal surgery for pituitary tumors.

### Strengths and limitations

The strength of this study is the relatively large sample size from a well-described national cohort. We were able to study a 10 day-postoperative period, including meticulous follow-up of the plasma sodium concentration. Our study also has several limitations, which have to be acknowledged. A prospective cohort study with standardized data collection would be more optimal to study the short-term and long-term effects of CDI, compared to the current retrospective design. Additionally, more detailed information could then be collected about fluid and desmopressin administration. The results of our study need to be regarded in this context. Due to the retrospective design, there were some missing data concerning pre-operative, perioperative (e.g. fluid balance) and postoperative management (e.g. dosage of dexamethasone administration, sodium supplementation in patients with hyponatremia, in some cases exact fluid balance). In 6 of the 81 patients with DI, the diagnosis of DI was based on documentation from the treating physician in the medical chart. In these patients, the diagnosis of DI had been made in the very first hours after surgery, while no retrospective data were available on plasma sodium concentrations directly perioperative. Furthermore, no information regarding urine specific gravity was collected. Urine specific gravity can be useful for the diagnosis of diabetes insipidus: polyuria with an increase in plasma osmolarity and urine osmolality dropping to below 300 mOsm/kg is suggestive for the presence of DI [24]. For these reasons, our results should be interpreted with care before causal interpretations are made. Larger prospective studies are needed to elucidate these possible relations, along with the question as to whether the severity of fluctuations in plasma sodium concentration and neurological consequences can be reduced by further centralization of care.

## Conclusion

Early postoperative CDI is frequently seen in children operated on a sellar or suprasellar tumor. Patients with early postoperative CDI and steep decline in plasma sodium concentration are at risk to develop an altered mental status and seizures in the first 10 days postoperative. Despite current high-level care, many outliers and fluctuations of plasma sodium concentration are still being observed in the postoperative period. Therefore, patients requiring surgery in the sellar or suprasellar region should be monitored in a highly experienced center. Possible improvement of outcome could be strived for by using multidisciplinary protocolized care for maintaining an adequate sodium and water balance.
